# Supplementary figures and images for: Uncovering in vivo biochemical patterns from time-series metabolic dynamics
Source: PLoS One. 2022 May 12;17(5):e0268394. doi: 10.1371/journal.pone.0268394 (PMC9098013; doi:10.1371/journal.pone.0268394)

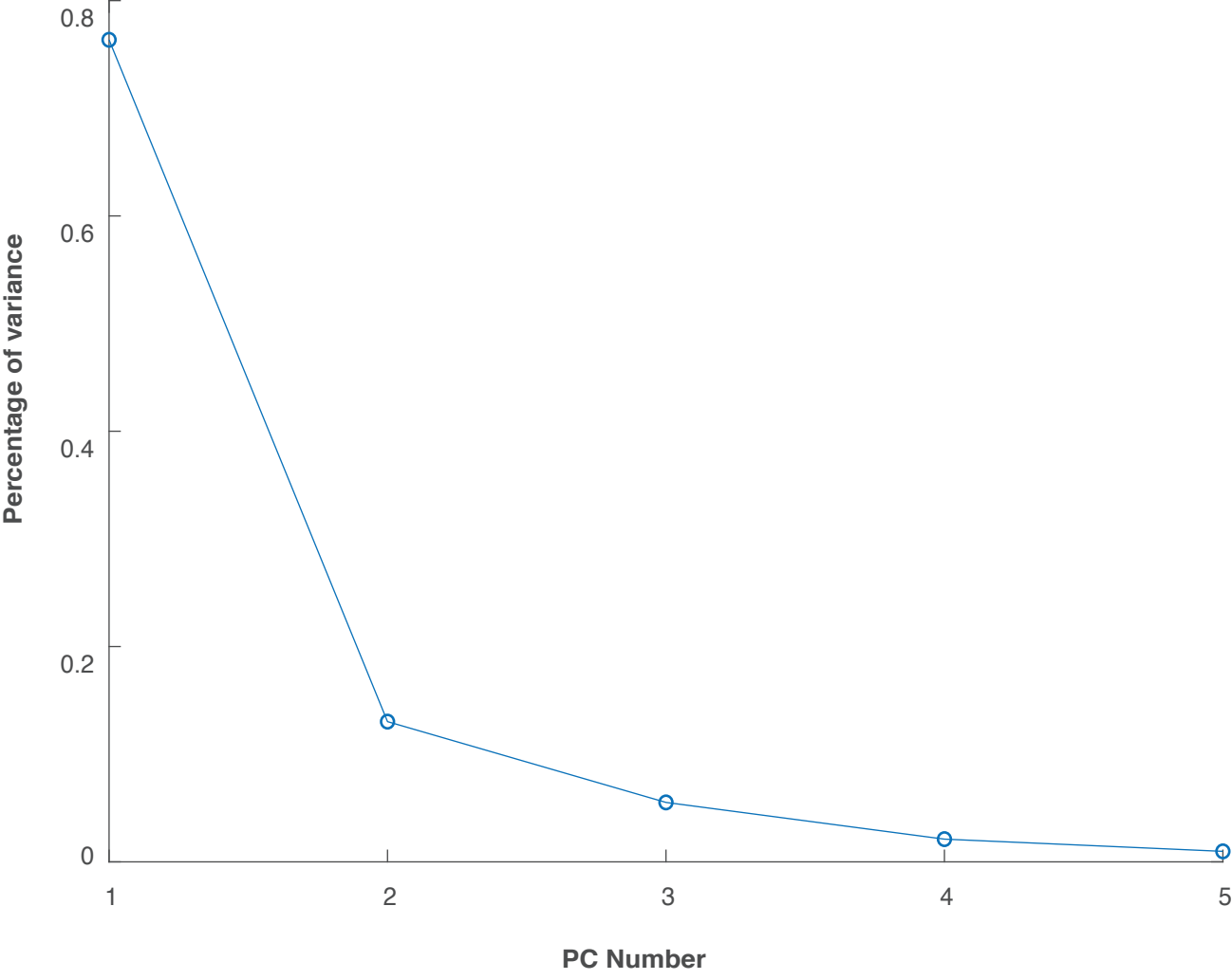

Supplement: S1 Fig — The X (Y) axis represents the number of PC (percentage of variance). The first two PCs, especially PC1, explain the most variance. The corresponding scores plot and eigenfunction can be found in Fig 2B. (PDF) [file pone.0268394.s001.pdf]

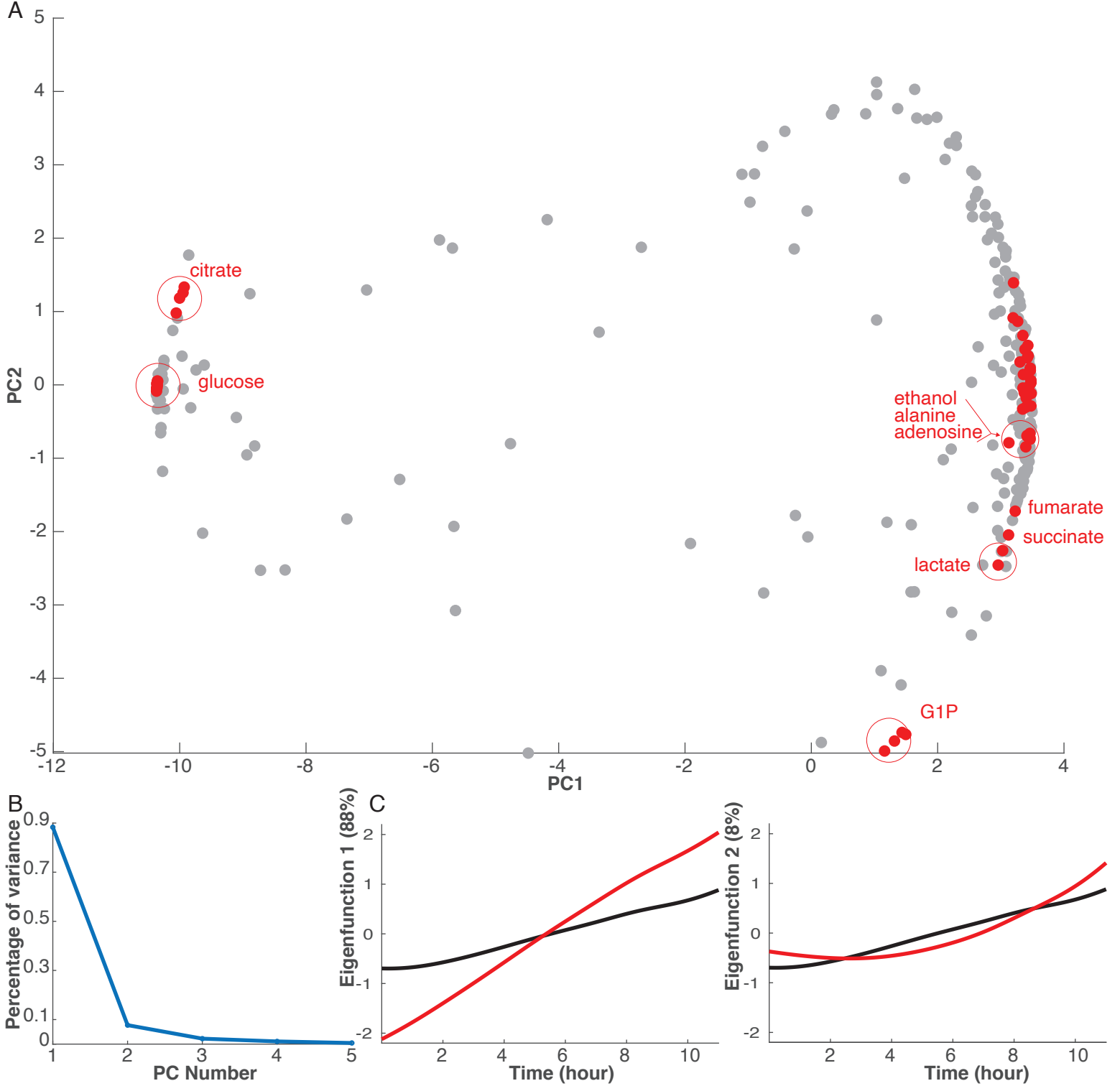

Supplement: S2 Fig — A: The first two PC dimensions are visualized, and dominant changing patterns are presented. Each point represents the time series of one NMR feature, and some of them are highlighted with compound annotations (red). The X (Y) axis represents scores for PC 1 (2). B: Percentages of explained variance are presented for the first few PCs. The X (Y) axis represents the number of PC (percentage of variance). The first two PCs, especially PC1, explain most variances. C: Eigenfunctions are plotted for PC1 and PC2. The middle black curve represents the mean time series; the red curve represents the effects of adding a fraction (square root of eigenvalue) of the corresponding eigenfunctions to the mean curve. The X (Y) axis represents time (value). The percentages of variance explained are presented in parentheses. NMR features were centered and scaled before the PCA analysis. Results for aerobic conditions can be found in Figs 2 and S1. (PDF) [file pone.0268394.s002.pdf]

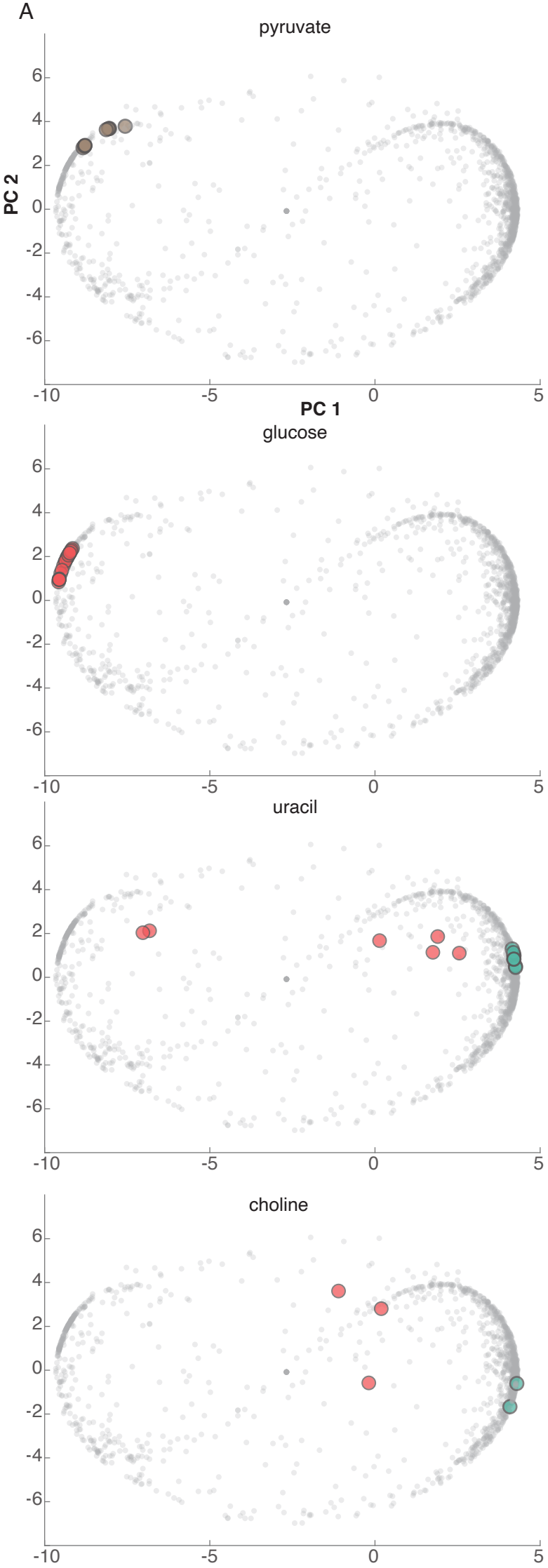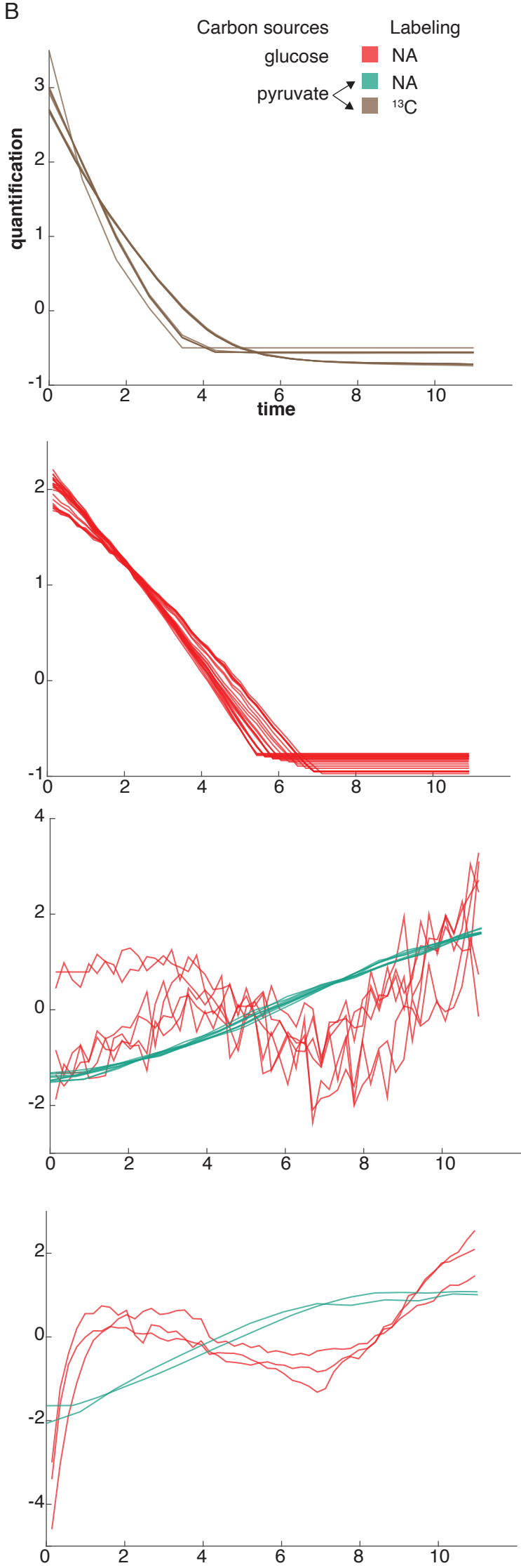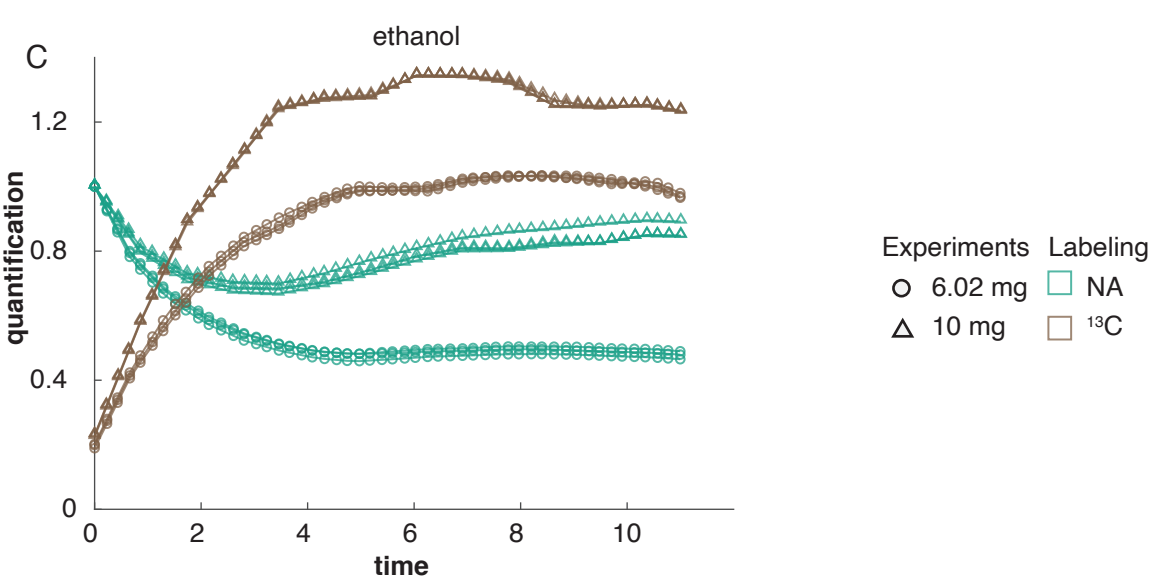

Supplement: S3 Fig — Two different carbon sources were compared: natural abundance glucose (3 experiments) and uniformly 13C-labeled pyruvate (2 experiments). The glucose experiments were all done at a high density (10 mg/63 μL), and the pyruvate experiments were done at low (6mg /63 μL, solid lines) and high (10 mg/63 μL, dashed lines) densities. The chemical features from the glucose experiments are shown in red. The 13C-labeled metabolites produced in the 13C-pyruvate experiments are shown in brown. The unlabeled metabolites produced in the 13C-pyruvate experiments are shown in green. A: FPCA score plot indicates the overall patterns of different compounds in each of these experiments. Each point represents one ridge in one sample. The small, grey points correspond to all the other ridges detected in these experiments. The X (Y) axis represents scores for PC 1 (2). The score plot was kept the same with different compounds highlighted. B: Time trajectory (hours) of the highlighted features from A. Each curve was centered and scaled for A and B. C: Detailed comparison of ethanol in the 13C-pyruvate experiments. Two experiments with different amounts of organisms are visualized by different shapes. 13C-labeled and unlabeled ethanol are distinguished by colors. Normalization was applied to make them comparable. Ridges of triplets around 1.1 ppm were used to quantify ethanol in C, and all tracked ridges were used in Fig 3A and 3B. (PDF) [file pone.0268394.s003.pdf]

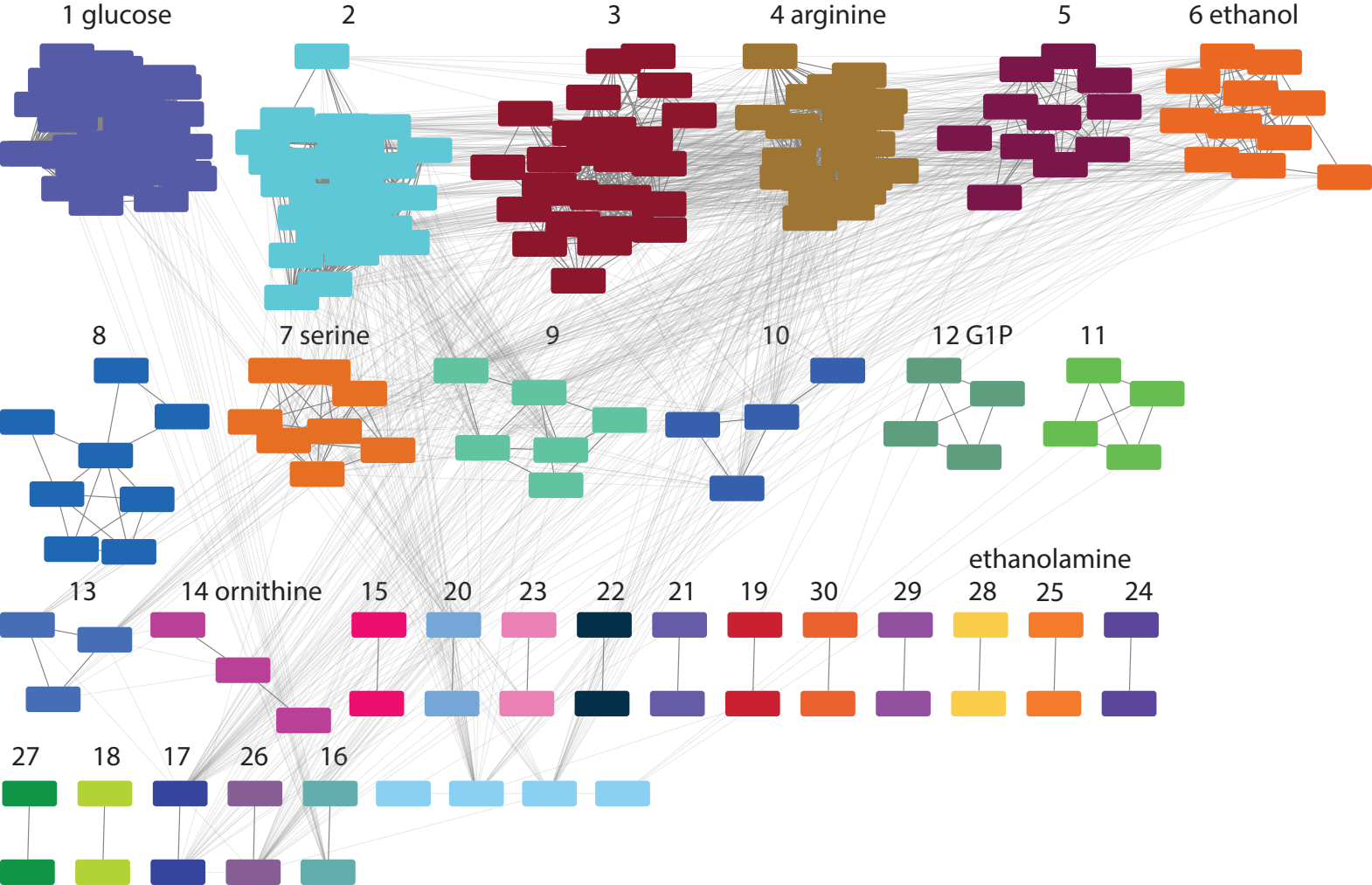

Supplement: S4 Fig — A correlation network was built upon time-series features in the glucose feeding experiments, and clusters were found (More details in Methods). Each cluster is highlighted by a different color and assigned one number. Single nodes are in blue. Details of specific clusters are shown in Fig 4. (PDF) [file pone.0268394.s004.pdf]

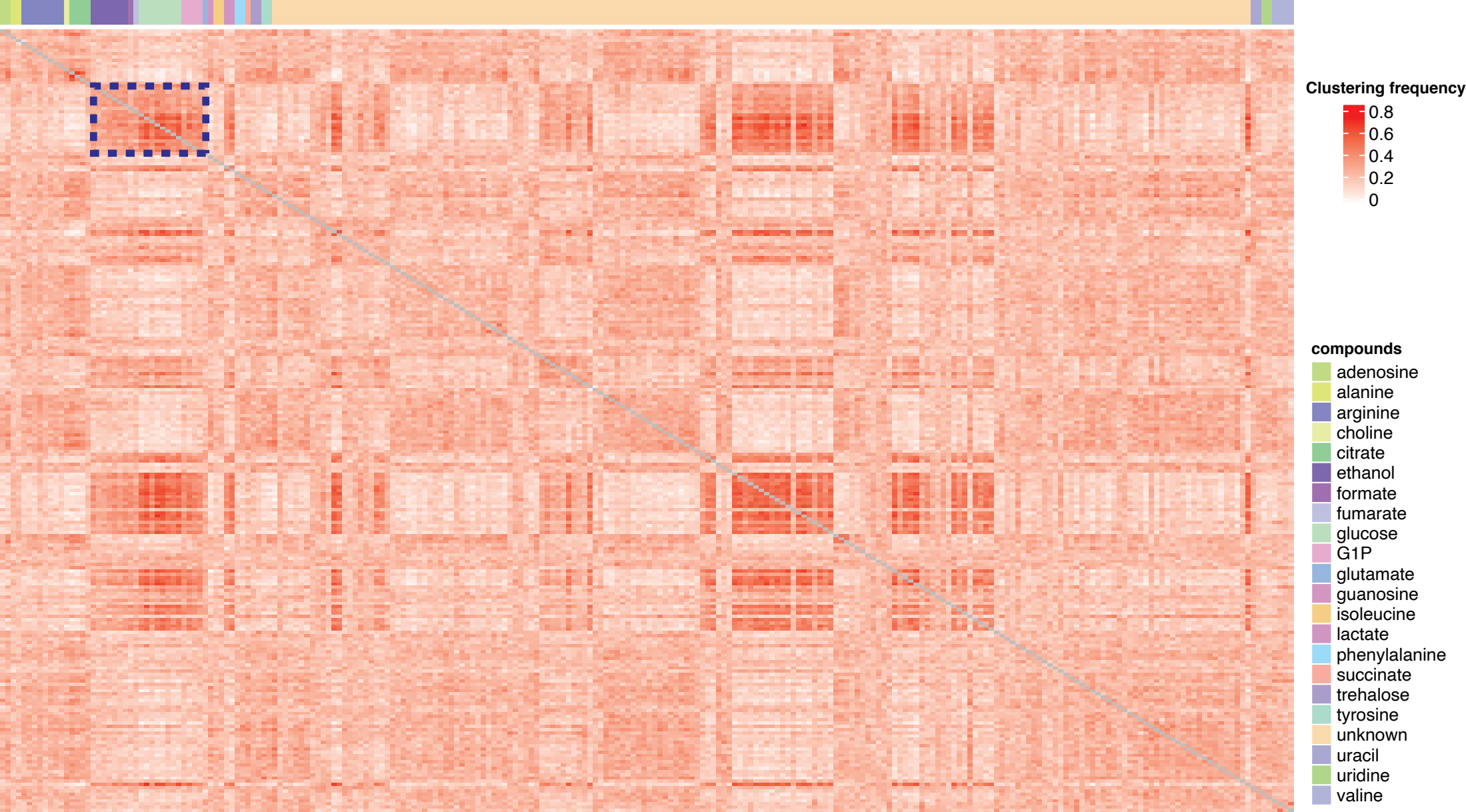

Supplement: S5 Fig — Colors in the heatmap represent relative frequencies that two NMR features share the same clusters in bootstrapping. Each row or column represents one feature. Red (white) indicates more (less) co-occurrences of the two features. The top bar indicates different compounds by colors. The dashed box highlights nodes of glucose, ethanol and G1P that are frequently presented in the same cluster. Diagonal values are not presented, as the same feature will always be in the same cluster. The bootstrapping results are also visualized in the network in Fig 5B. (PDF) [file pone.0268394.s005.pdf]

A

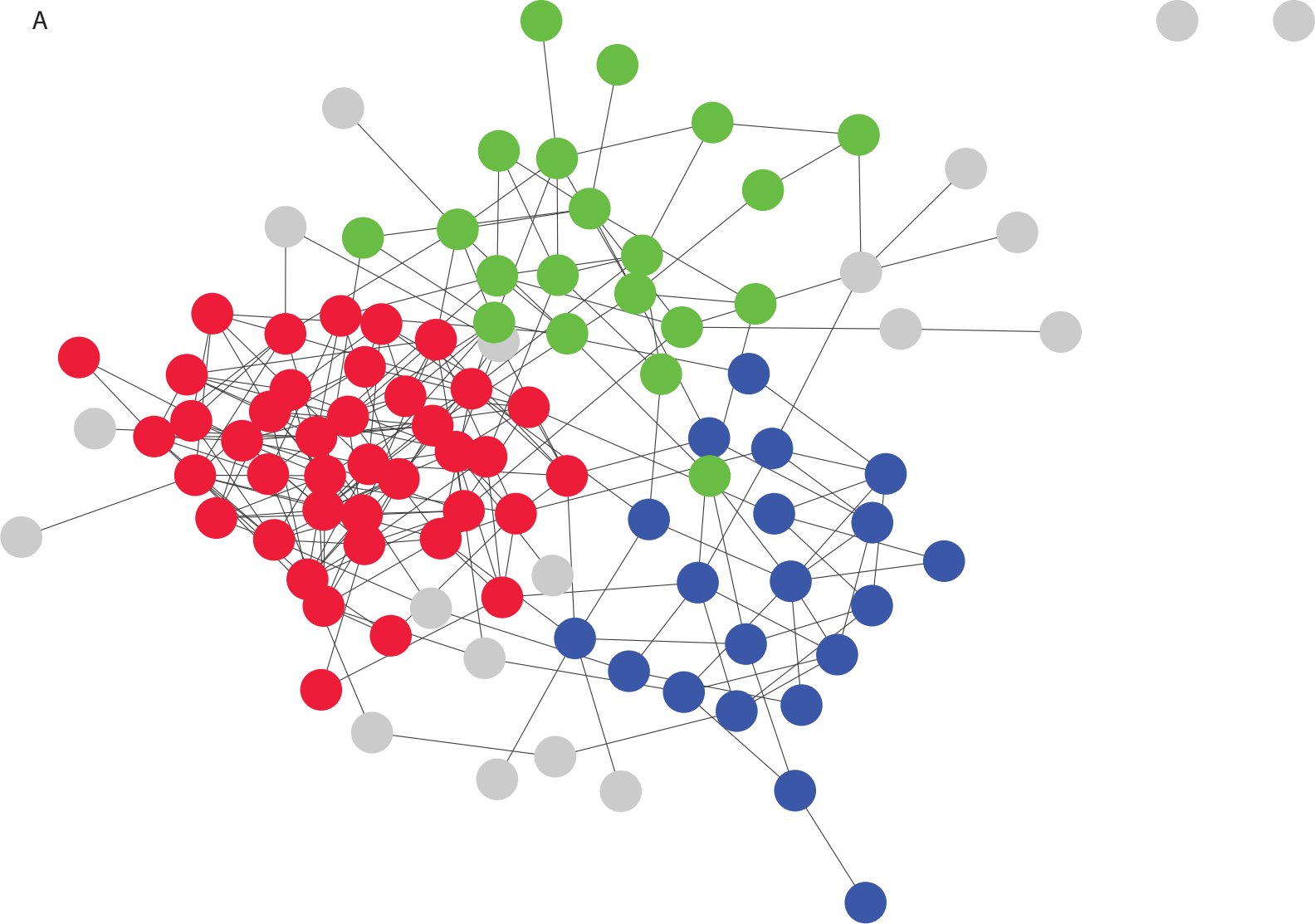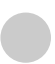

B

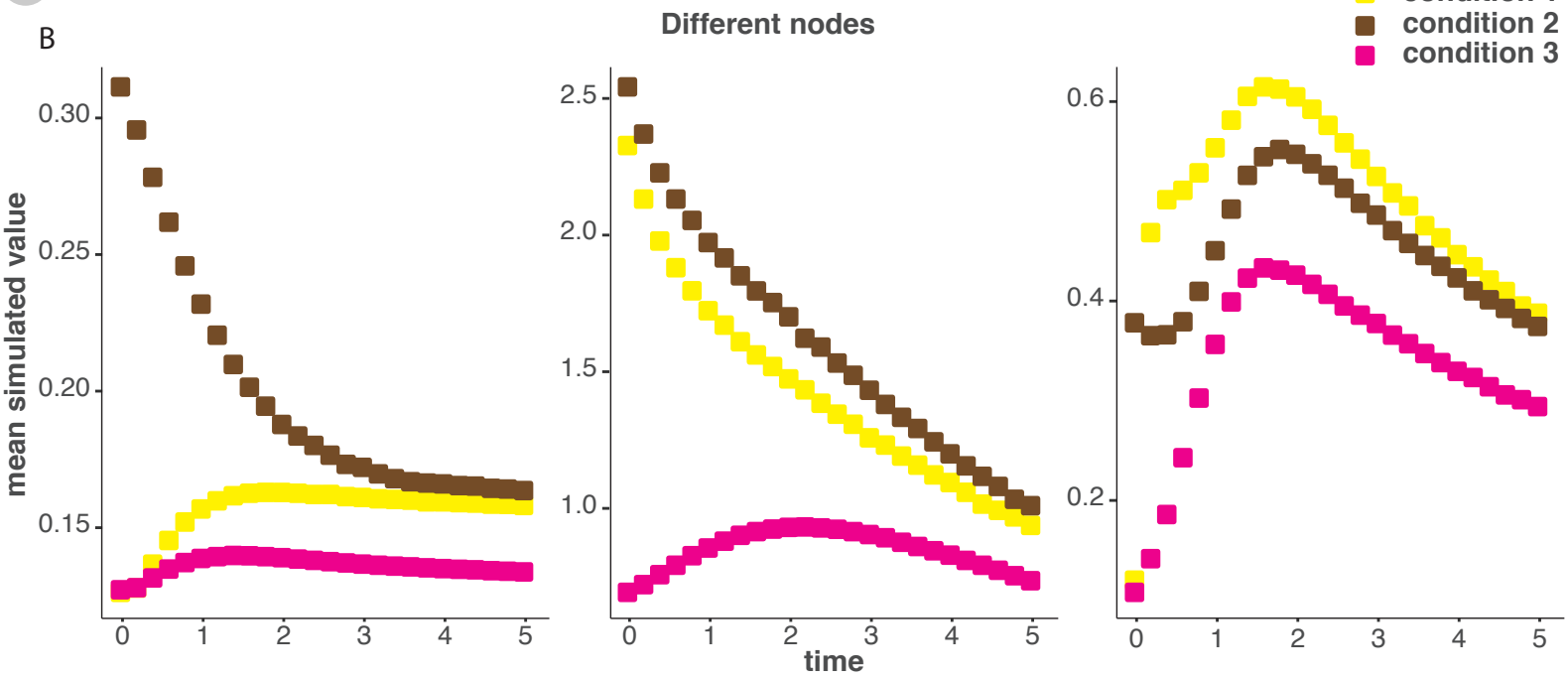

Supplement: S6 Fig — One example random network and corresponding dynamics are presented. A: The random network (with 100 nodes) was simulated with clusters. Red, green and blue nodes indicate three simulated clusters where internal links are denser than inter-cluster links. Gray nodes do not belong to any clusters. B: Time-series dynamics were simulated for each node under different initial conditions. The X (Y) axis indicates time (mean simulated value). Trajectories of three different nodes under three different conditions are presented. The mean value was calculated from three different replicates with the same nodes and conditions but different random noise. More detail on random networks and dynamics simulation can be found in Methods and S1 File. (PDF) [file pone.0268394.s006.pdf]

A

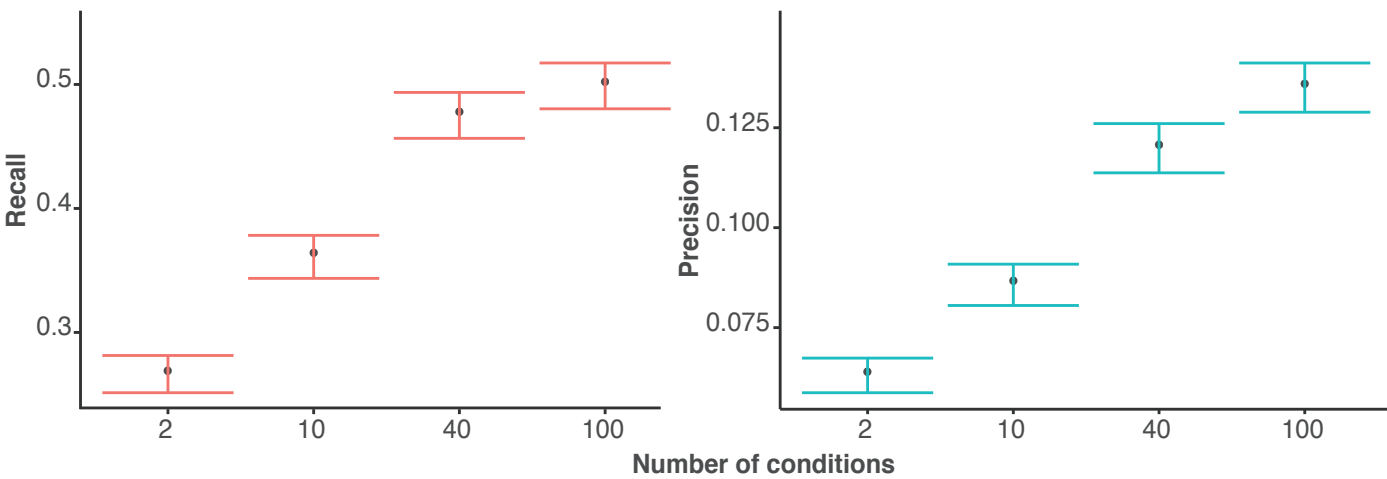

B

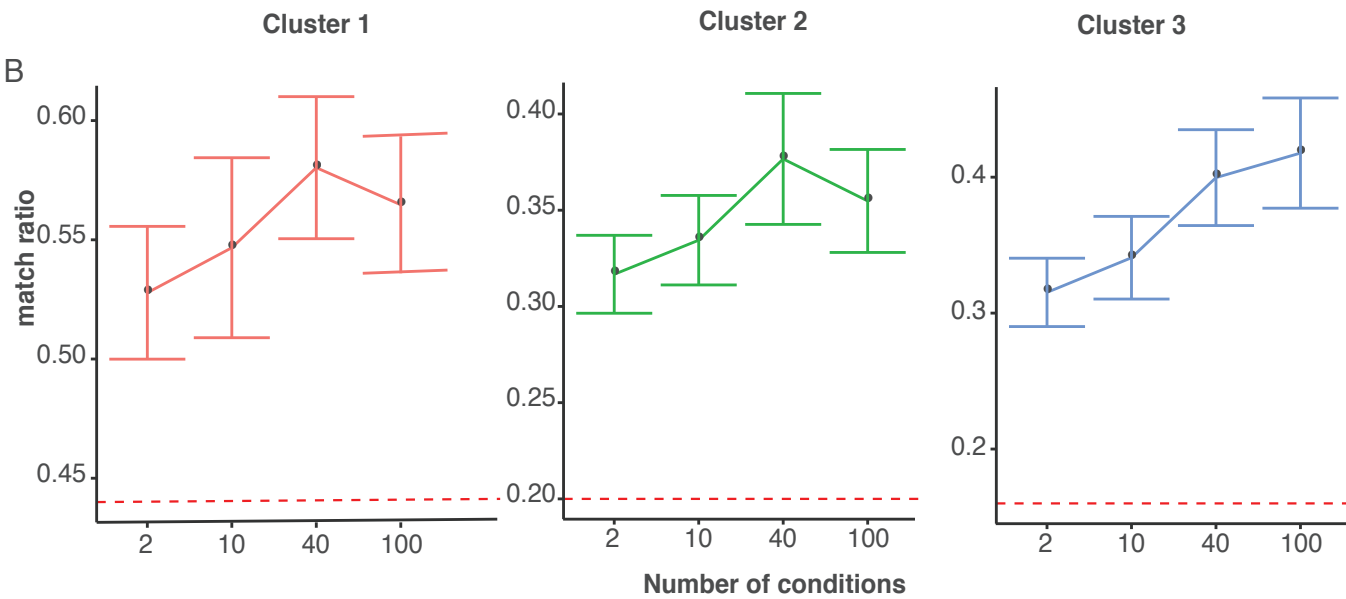

Supplement: S7 Fig — Our workflow was evaluated on a simulated benchmark dataset with partial observation. In the random network, each node (100 in total) represents one compound, and each cluster is a set of compounds with denser inner cluster connections. Time dynamics were simulated based on the networks through ODEs under different initial conditions. A subset of the time-series features was observable and clustered (More details in Methods). The performance in estimating edge and recovering clusters was evaluated. A: Recall and precision (Y-axis) for edge estimation are presented under different numbers of initial conditions (X-axis). Edge estimation improves with more conditions. B: The performance in cluster recovering is presented for the three clusters under a different number of conditions. The X (Y) axis represents the number of conditions (match ratio). The match ratio is the proportion of nodes from the matched real cluster in the best-recovered cluster (More details in Methods). The red dotted lines indicate the match ratio of a random group of nodes (baseline). The estimated clusters can recover more nodes from real clusters, and the performance improves with more conditions. The error bars represent two standard errors calculated from the simulation and reconstruction of 59 random networks. Simulated random networks and example time series can be found in S6 Fig. Simulation-based evaluation with redundant signals can be found in S8 Fig. The performance on an experimental dataset can be found in Figs 5B and S5. (PDF) [file pone.0268394.s007.pdf]

A

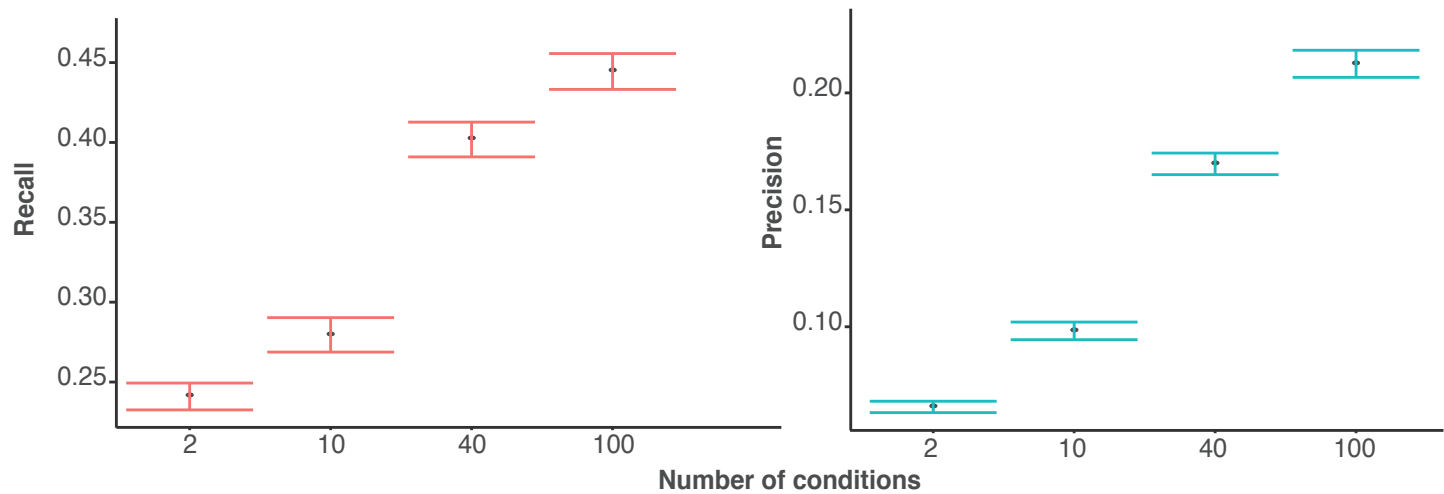

B

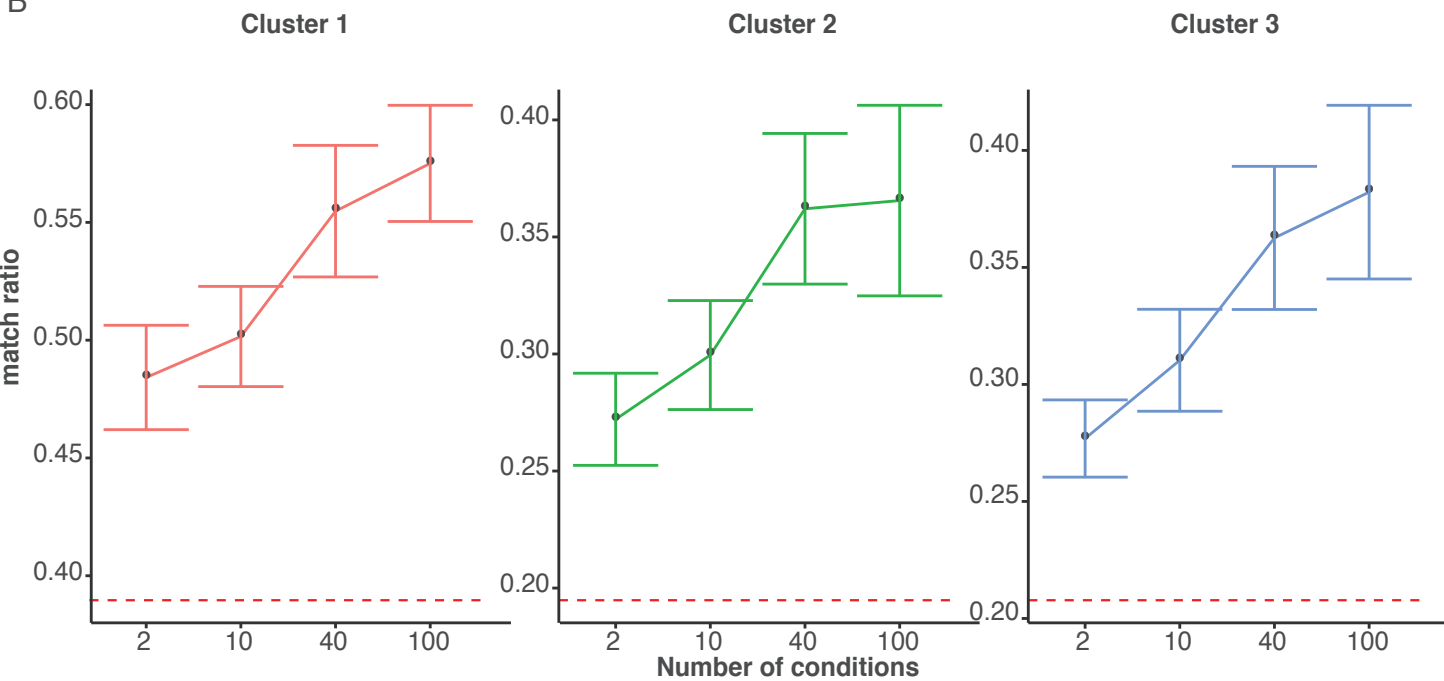

Supplement: S8 Fig — Our workflow was evaluated on a simulated benchmark dataset with partial observation and redundant signals. In the random network, each node (100 in total) represents one compound, and each cluster is a set of compounds with denser inner cluster connections. Time dynamics were simulated based on the networks through ODEs under different initial conditions. Time-series features were also expanded and scaled by random factors as an analogy of multiple peaks corresponding to the same compound in NMR. A subset of the time-series features was observable and clustered (More details in Methods). The performance in estimating edge and recovering clusters was evaluated. A: Recall and precision (Y-axis) for edge estimation are presented under different numbers of initial conditions (X-axis). Edge estimation improves with more conditions. B: The performance in cluster recovering is presented for the three clusters under a different number of conditions. The X (Y) axis represents the number of conditions (match ratio). The match ratio is the proportion of nodes from the matched real cluster in the best-recovered cluster (More details in Methods). The red dotted lines indicate the match ratio of a random group of nodes (baseline). The estimated clusters can recover more nodes from real clusters, and the performance improves with more conditions. The error bars represent two standard errors calculated from the simulation and reconstruction of 59 random networks. Simulated random networks and example time series can be found in S6 Fig. Simulation-based evaluation with no redundant signals can be found in S7 Fig. The performance on the experimental dataset can be found in Figs 5B and S5. (PDF) [file pone.0268394.s008.pdf]

# G1P Spike experiment

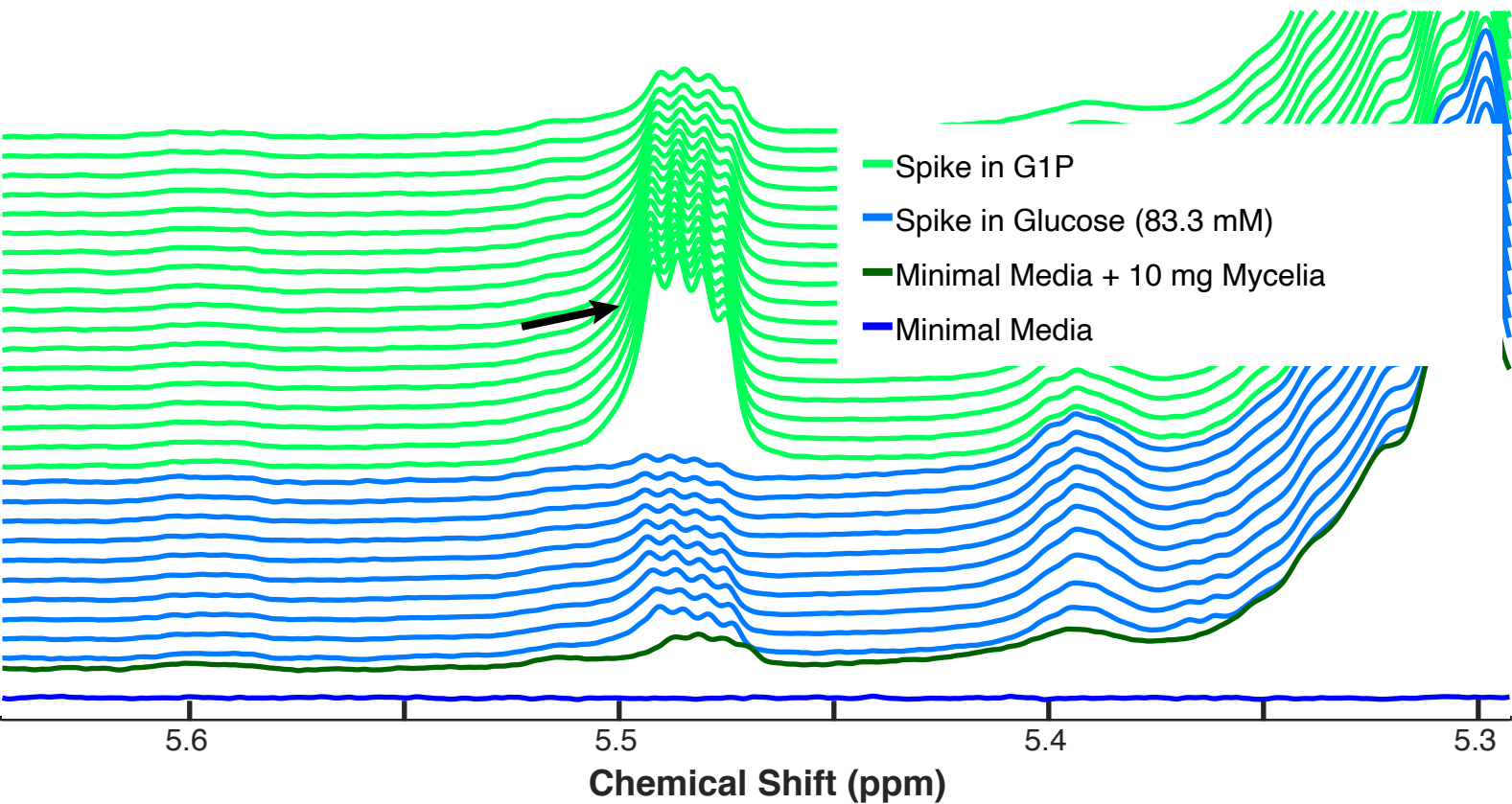

Supplement: S9 Fig — Multiple contrasting experiments were collected through time: minimal media [2], minimal media and 10 mg mycelia, spiking glucose in the culture, and spiking G1P in the culture. The X-axis represents chemical shift. The arrow shows the G1P peaks, which increased after G1P spiking. (PDF) [file pone.0268394.s009.pdf]
