# Supplementary material for: Uncovering in vivo biochemical patterns from time-series metabolic dynamics
Source: PLoS One. 2022 May 12;17(5):e0268394. doi: 10.1371/journal.pone.0268394 (PMC9098013; doi:10.1371/journal.pone.0268394)
Supplement: S1 File — Edge and cluster estimation was evaluated on a simulated benchmark dataset. (DOCX) [file pone.0268394.s011.docx]

**Background of the simulation test**

Our work also adds to topology estimation based on time-series data [1, 2]. Different approaches in topology (edge) and parameter estimation in biological dynamical systems have been compared in the DREAM7 competition [1]. This early benchmarking study focused on networks with few hidden connections and limited topological diversity. The experimental dataset provides a useful but limited evaluation of network construction and cluster. Collecting time series data with multiple perturbation conditions is also expensive.

Here, we simulated benchmarking datasets with a considerable number of unknown connections, extensive partial observation and signal duplication, which better represents exploratory metabolomic experiments. Partial observation represents the absence of data for many metabolites in time-series profiling experiments [3-5]. Duplicated signals arise in NMR spectra, where the same compound produces multiple peaks.

**Performance evaluation based on benchmarking dataset**

Metabolites (nodes) are connected through reactions and regulation (edges), and there are multiple functional clusters in biochemical pathways. To resemble the pathway architecture, we simulated random networks with clusters, and nodes were more frequently connected within clusters (S6A Fig; More details in Methods). The three clusters represent groups of nodes related to three different functions. From the random network, time-series dynamics were simulated based on different initial conditions (S6B Fig), and they were the benchmark input.

Edge estimation improves with more experimental conditions (S7A and S8A Figs). In the simulation with partial observation and redundant signals (S8A Fig), precision and recall improve monotonically with more conditions. For dynamics generated from unknown network topology, recall (precision) can achieve around 45% (20%) with 100 conditions. Even with a low number of conditions, about 25% of real edges can be recovered though precision is low. As a comparison, in totally random cases, recall and precision should be about 2%. For the simulation with no redundant signals (S7A Fig), there are similar patterns in precision and recall though the improvement seems to plateau at around 100 conditions. Experimental measured metabolic data (e.g., NMR) have redundant signals for the same compounds so should still improve with more conditions.

The performance of recovering functional clusters also improves with more conditions (S7B and S8B Figs). The match ratio represents the proportion of nodes from the matched real cluster in the best recovered cluster. The ratios improve with more conditions for both simulations, even though the variance is still high with our number of simulation samples (59). The pattern is consistent for different cluster sizes, and they are all significantly higher than the random guess (Wilcox t-test p<0.01). For a cluster with 20% of nodes (total 100 nodes) and 100 conditions, on average more than 35% nodes of the best cluster are from the real cluster (S8B Fig), and those recovered real nodes represent around 40% of the real cluster.

**Reference:**

1. Meyer P, Cokelaer T, Chandran D, Kim KH, Loh PR, Tucker G, et al. Network topology and parameter estimation: from experimental design methods to gene regulatory network kinetics using a community based approach. Bmc Systems Biology. 2014;8.

2. Al-Omari A, Griffith J, Caranica C, Taha T, Schuttler HB, Arnold J. Discovering Regulators in Post-Transcriptional Control of the Biological Clock of Neurospora crassa Using Variable Topology Ensemble Methods on GPUs. Ieee Access. 2018;6:54582-94.

3. Judge MT, Wu Y, Tayyari F, Hattori A, Glushka J, Ito T, et al. Continuous in vivo Metabolism by NMR. Frontiers in Molecular Biosciences. 2019;6(26).

4. Koczula KM, Ludwig C, Hayden R, Cronin L, Pratt G, Parry H, et al. Metabolic plasticity in CLL: adaptation to the hypoxic niche. Leukemia. 2016;30(1):65-73.

5. Link H, Fuhrer T, Gerosa L, Zamboni N, Sauer U. Real-time metabolome profiling of the metabolic switch between starvation and growth. Nature methods. 2015;12(11):1091-7.
