# Supplementary material for: Uncovering in vivo biochemical patterns from time-series metabolic dynamics
Source: PLoS One. 2022 May 12;17(5):e0268394. doi: 10.1371/journal.pone.0268394 (PMC9098013; doi:10.1371/journal.pone.0268394)
Supplement: Attachment — Submitted filename: response.docx [file pone.0268394.s014.docx]

Reviewer #1: This study involves the development of a new analytical workflow to visualise complex NMR-based metabolomic data from fungus cultures. The approach has the potential to reveal new metabolic signatures and speed processing so is widely but the manuscript is written in a technical manner lacking key details that would make it approachable to a more general reader. Better annotation of Figures (particularly the supplemental information) and restructuring of manuscript (clear separation of legends, results narrative and discussion commentary) would also improve readability and accentuate the impact for the general audience of this journal.

Thank you for your comments. We added some clarification content and restructure the manuscript to improve accessibility to general audiences.

I appreciate that the focus here is on the data processing but the fungal cells have a relatively simple metabolome. To validate the downstream impact of such an approach it would be reassuring to see analysis of additional data from a complex cell line or human cell system where different cells within a mixture may exhibit metabolic variation. Alternatively data from Neurospora cultured with a metabolic stressor/altered culture medium conditions to check that data arising make biological sense

We collected a new *N. crassa* dataset with uniformly ^13^C-labeled pyruvate as carbon sources (Results 2.3). Consistent patterns were found in pyruvate consumption and production of other compounds (Figs 3 and S3). Different patterns in ^13^C and unlabeled ethanol indicate different metabolic processes of consuming external carbon sources and internal metabolism. We have done some studies in human cells, but they are much more delicate than *N. crassa* and would not be amenable to 12+ hour measurements.

The analysis is based on a published data set generated using cultures under aerobic and anerobic conditions. Even though the data is preexisting it would be important to provide more information on the approach in the methods section (eg source of fungus and cultural conditions/densities, metabolite extraction procedure, annotation method) since fungal life stage will impact on outcome under different conditions. Similarly the advantage here is the ability of the analysis to track analytes over time and pick up peaks unassigned by other approaches. Thus it may be worth including a schematic diagram or summary of the analytical workflow to highlight the key advantages of the system.

We added a brief section describing the experiments (Methods 4.1). We added a diagram (Fig 1) to describe the computational workflow and highlight some advantages (Results 2.1). More details can be found for experimental techniques [1] and feature extraction [2].

Figure 1 – the stack plot here is a nice way to visualize the temporal change in spectra. Would it be possible to annotate some of the key peaks (glucose, ethanol, G1P etc) and show a similar composite image for spectra collected under anaerobic conditions? This would help with assessment of the latter Figures. In particular it would be helpful to see the arginine plot on this figure so that it can be compared with the output in Figure 2

Thank you for this suggestion. The suggested figure has already been published and we point the reviewer to figure 3 in the original CIVM-NMR publication [1]. In that figure, a much broader spectral range was annotated, including glucose, ethanol and G1P. A mirror plot was constructed as the reviewer suggested to compare aerobic and anaerobic conditions. Arginine peaks can be found from 1.6 ppm to 2 ppm, which exist in both aerobic and anaerobic conditions.

Is there choline in the media used to grow the cells?

There is no choline in the media. Vogel’s is a chemically defined *Neurospora* media. It contains essential vitamins and minerals, water, and a carbon source (glucose or pyruvate, in this case). We only modified this media by removing citrate and adding DSS (Sodium trimethylsilylpropanesulfonate), which is a commonly used NMR referencing compound [1].

The ridges within the trace form the basis of the cluster analysis. Does this introduce a bias for detection of more abundant metabolites even though aim is to maximize visibility by scanning over a timeframe?

We agree that if the compound has a low concentration and is overlapped with more abundant compounds, the annotation and quantification will be hard by RTExtract or by experts. We are working on a separate spectral decomposition method to solve this problem. For low concentration compounds with no direct overlap, they will still form a ridge through time and can be tracked even if the signal to noise is bad (S2 Fig in the RTExtract paper) [2]. Additionally, we can track low-intensity peaks for the few time points they clearly appear and so, increase the number of quantifiable spectral peaks. In each CIVM-NMR experiment, we can track about 300 ridges and many compounds in central metabolism can be quantified.

Could the approach be combined with isotopic metabolic tracer analysis using labelled precursors to confirm that the hypothesized fluxes (Fig 4) are detected in the time resolved analysis? Otherwise the discussion of this data is a little speculative.

Yes.

To address this comment, we collected an experiment with uniformly ^13^C labeled pyruvate as carbon sources and measured both natural abundance and ^13^C species (See response to Q1 and Results 2.3). FPCA effectively separated the peaks of the same compounds under different carbon sources and densities, providing a descriptive, low-dimensional profile of each experiment. Importantly, this experiment revealed distinct pools of either unlabeled or ^13^C-labeled ethanol. A similar phenomenon was observed in the original CIVM-NMR paper [1] and certainly merits future investigation as it suggests different pathways or regulation. We feel this experiment has added to our study substantially.

For the flux in central energy metabolism (Fig 6A), the PGM2 mutant experiment (Discussion 3.1 paragraph 2) will be more appropriate as validation. The regular flux from glucose to ethanol, alanine, and lactate has already been shown in the S7 Figure in our previous publication [1]. Our novel discovery focuses on the role of G1P in central energy metabolism. The main hypothesis is that the metabolic associations with G1P (in the FN network) depend on phosphoglucomutase and its reversible connection to glycolysis (Discussion 3.1 paragraph 2). If the hypothesis is true, the corresponding mutants PGM2 should have much fewer FN connections with G1P. The experiment will be wild-type compared with PGM2, and a few medium conditions will be needed on each side. We are still collecting data for this experiment.

Much useful information is supplied in supplemental figures but these are barely referred to or describes in places. File S1 is a word document describing benchmarking. Not sure how this works as a supplemental information source?? Should it be badged as supplemental methods??

Thank you very much for this suggestion. We modified the main text to refer to those files more frequently and clearly. File S1 describes the result of benchmarking test, and the corresponding method is described in the main text (Discussions 4.7 and 4.8).

Reviewer #2: This manuscript describes an approach to improve identification of metabolites in longitudinal metabolic profiles using correlation networking. The approach is sound and this is an area of intensive developments.

Thank you for your comments. Approach to annotation is one important part of this manuscript.

The document is written in a manner that is difficult to follow. Some sections are very detailed when not really necessary while the introduction is very sparse. The draft would greatly benefit from a Figure 1 with a proper explanation of the whole procedure that would guide the reader and also help the structure of the text. For instance when discussing the expansion of the annotation I found confusing the part where annotations are found because of correlation or because of look up into COLMAR. I understand that a correlation STOCSY or even covariance NMR approach produces a TOCSY-like spectra that can be compared with real spectra, but in the text it is not clear what type of data is input of what process. Is the CN used to build a spectra as an input into COLMAR or is the network directly compared with other networks obtained from spectra? As this is not clear, it makes it difficult to understand what data is annotated using COLMAR and what data is annotated by CN prior to COLMAR. Page 9, lines 188 to 196 are very difficult to follow.

We assigned a section to describe the whole workflow (Fig 1; Results 2.1) and modify the introduction for clarification.

We updated the CN section (Results 2.4) to clarify. Here we presented a new annotation process: building CN (correlation network) from CIVM-NMR ridges, finding clusters in the network and automatically searching for chemical shifts of each cluster in GISSMO and COLMAR 1D database (Methods 4.4). The traditional process includes selecting time points for sample extraction, conducting 2D NMR experiments and checking with COLMARm manually. The new process doesn’t need 2D experiments, extraction, and mapping from 2D matches to CIVM-NMR spectra, which two are often different in peak intensity and overlap patterns (Results 2.4 paragraph 2). Our new method can uncover some new compounds (serine, ethanolamine, and ornithine; Fig 4; Results 2.4 paragraph 4) and supply more quantifiable peaks for existing annotations (Results 2.4 paragraph 3). CN is presented as a quick annotation process directly based on CIVM-NMR measurements rather than a replacement of the traditional 2D NMR approach.

The next step is also described in a confusing manner, is the functional network used to improve the annotation or is the previous clustering used to improve the outcome of the functional networking? I guess the former, but then the authors fails (in my opinion) to discuss how this form of clustering is different, or give different results, than the correlation network, as both are fed with the same information, except for the derivation that can be seen as a pre-processing step as in many other analytical pipelines. Sentences as in page 13 lines 279: Multiple clusters were recovered... are not precise at describing the outcome. Such sentences are found a lot in the manuscript.

We add the first result section to clarify (Fig 1; Results 2.1; Fig 1). We correct several sentences to improve precision.

In parallel to CN for compound annotation, FN is a process to infer *in vivo* biochemical processes and regulation (Fig 1). The reviewer is correct that the same information is fed, but the model searching process is different for the different goals. CN uses the correlation of peak intensity to find metabolite annotations (Results 2.4 paragraph 1 and Methods 4.4) as different peaks of the same compound should be highly correlated. FN searches for metabolic-reaction-like models where changing rates of one metabolite depend on the concentrations of others through a recently published statistical method CausalKinetiX (Results 2.6 paragraph 1, Methods 4.6) [3]. In the manuscript, it searched for the models where derivative of one ridge on the left side and a few ridges on the right side (equation 2). 1^st^ derivative is used to calculate the rate. Other differences exist as CausalKinetiX searched for stable and predictive statistic models instead of calculating distance metrics (e.g., correlation). Detailed information on CausalKinetiX can also be found here [3].

I therefore suggest that the authors carefully worked on the structure of their document to better herald their interesting approach. As it is I would not recommend publication.

Thank you for consideration. We have undertaken a substantial rewriting process to address this concern.

Reference:

1.	Judge MT, Wu Y, Tayyari F, Hattori A, Glushka J, Ito T, et al. Continuous in vivo Metabolism by NMR. Frontiers in Molecular Biosciences. 2019;6(26).

2.	Wu Y, Judge MT, Arnold J, Bhandarkar SM, Edison AS. RTExtract: time-series NMR spectra quantification based on 3D surface ridge tracking. Bioinformatics. 2020;36(20):5068-75.

3.	Pfister N, Bauer S, Peters J. Learning stable and predictive structures in kinetic systems. Proceedings of the National Academy of Sciences of the United States of America. 2019;116(51):25405-11.
